# Glucosinolate-rich broccoli sprouts protect against oxidative stress and improve adaptations to intense exercise training

**DOI:** 10.1016/j.redox.2023.102873

**Published:** 2023-09-03

**Authors:** M. Flockhart, L.C. Nilsson, E.N. Tillqvist, F. Vinge, F. Millbert, J. Lännerström, P.H. Nilsson, D. Samyn, W. Apró, M.L. Sundqvist, F.J. Larsen

**Affiliations:** a Department of Physiology, Nutrition and Biomechanics, Åstrand Laboratory, The Swedish School of Sport and Health Sciences, Stockholm, Sweden; b Linnaeus Centre for Biomaterials Chemistry, Linnaeus University, Kalmar, Sweden; c Department of Chemistry and Biomedicine, Linnaeus University, Kalmar, Sweden; d Department of Laboratory Medicine, Clinical Chemistry, Örebro University Hospital, Örebro, Sweden; e School of Medicine, Faculty of Medicine, Örebro University, Örebro, Sweden

## Abstract

Oxidative stress plays a vital role for the adaptive responses to physical training. However, excessive oxidative stress can precipitate cellular damage, necessitating protective mechanisms to mitigate this effect. Glucosinolates, found predominantly in cruciferous vegetables, can be converted into isothiocyanates, known for their antioxidative properties. These compounds activate crucial antioxidant defence pathways and support mitochondrial function and protein integrity under oxidative stress, in both Nrf2-dependent and independent manners. We here administered glucosinolate-rich broccoli sprouts (GRS), in a randomized double-blinded cross-over fashion to 9 healthy subjects in combination with daily intense exercise training for 7 days. We found that exercise in combination with GRS significantly decreased the levels of carbonylated proteins in skeletal muscle and the release of myeloperoxidase into blood. Moreover, it lowered lactate accumulation during submaximal exercise, and attenuated the severe nocturnal hypoglycaemic episodes seen during the placebo condition. Furthermore, GRS in combination with exercise improved physical performance, which was unchanged in the placebo condition.

## 1 Introduction

Short periods of intense exercise training have been shown to induce substantial metabolic and circulatory adaptations that can improve health and physical performance. At the same time, exercise training imposes a significant organismal challenge with increased oxidative and energetic stress [1] that needs to be managed through homeostatic mechanisms. One such mechanism is the transcription factor nuclear factor erythroid 2-related factor 2 (Nrf2) which activates critical endogenous antioxidant defence pathways and maintain mitochondrial function and protein integrity during oxidative stress [2]. Also, Nrf2 is crucial in coordinating the adaptive response to exercise training [3] (see Table 1).

**Table 1:** Overview of the training protocol and average power output during each condition.

Day	Training	Avg watt GRS	Avg watt placebo
1	5 × 4 min @ 90–95% VO_2_max	204±29	205±30
2	5 × 4 min @ 90–95% VO_2_max + 2 × 30sek sprint	209±28	210±28
3	5 × 8min @ 85–90% VO_2_max	187±25	186±17
4	5 × 4 min @ 90–95% VO_2_max + 3 × 30sek sprint	213±32	216±36
5	5 × 8min @ 85–90% VO_2_max	193±27	194±25
6	5 × 4 min @ 90–95% VO_2_max + 4 × 30sek sprint	215±34	216±36
7	5 × 4 min @ 90–95% VO_2_max + 5 × 30sek sprint	212±32	209±26

Lack of Nrf2 has been shown to negatively affect skeletal muscle function after exercise in aged mice [4] and it also causes a faster decline in physical performance and muscle mass with age [5]. Acute exercise seems to increase mRNA of Nrf2 in both mice and humans as a response to oxidative stress. Although moderate exercise activates Nrf2, periods of excessive exercise have been shown to decrease Nrf2 expression and impair mitochondrial respiration with a simultaneous loss of metabolic adaptations and induce disturbances in glucose control [6].

Extensive research has demonstrated that the administration of direct antioxidants, such as vitamin C or E, during periods of exercise training may hamper the adaptive responses associated with training under certain circumstances [7]. These findings suggest that exercise-induced oxidative stress could exert advantageous signaling effects, contributing to the beneficial adaptations observed in response to exercise. Similarly, a cellular environment subtly skewed towards pro-oxidant conditions may be associated with improved adaptations to exercise training [8].

Expanding on the interplay between oxidative stress and exercise adaptations, isothiocyanates deserve attention. Isothiocyanates are a group of chemicals derived from glucosinolates found in plants in the *Brassica* genus that includes broccoli, kale, cauliflower, rocket, etc. Upon chewing or mechanical processing, glucosinolates become hydrolysed to isothiocyanates by the enzyme myrosinase. Although, more than 100 isothiocyanates have been identified to date and several have Nrf2-activating and other potent properties, the by far most studied isothiocyanate is sulforaphane. In addition to being potential Nrf2-activators, isothiocyanates also have Nrf2-independent properties, including inhibition of mitochondrial fission [9] and the mTOR pathway [10].

Isothiocyanates are electrophiles and function dually as pro-oxidants and antioxidants, much like exercise. They can disrupt cellular functions by forming covalent adducts, demonstrating acute pro-oxidant effects [11]. Through this transient oxidative stress, in the longer term, they enhance cellular antioxidant defenses and upregulate phase II detoxification enzymes [12]. This balance makes isothiocyanates compelling therapeutic candidates for conditions related to oxidative stress. Notably, isothiocyanates and particularly sulforaphane, have repeatedly been shown to stimulate endogenous antioxidant systems through Nrf2 activation in animal models following treatment periods typically spanning several days [13]. Intriguingly, studies have demonstrated an acute decrease in blood glutathione alongside with reductions in its precursors, glutamine and cysteine, in the hours following the ingestion of fresh isothiocyanate-rich broccoli sprouts [14]. This pattern suggests an initial rise in oxidative stress following GRS intake, which is subsequently counteracted by enhanced activation of endogenous antioxidants. To date, only a few studies have tested the effects of isothiocyanate administration in combination with exercise. Wang et al. found that mice receiving sulforaphane before exercise in hypoxia had higher expression of the lactate transporter MCT-1 and higher lactate dehydrogenase activity together with an increased running capacity [15]. Similarly, mice receiving an intraperitoneal dose of sulforaphane showed upregulated Nrf2 expression and improved running capacity, an effect absent in Nrf2-deficient mice [16]. Furthermore, young men receiving sulforaphane supplement for two weeks had higher mRNA expression of the Nrf2-target NQO1 in peripheral blood mononuclear cells and experienced less muscle soreness after a bout of intense weight training exercise [17]. In a placebo controlled cross-over study, young men received 30 mg of sulforaphane daily for 4 weeks and performed heavy resistance exercise sessions. Both creatine kinase and interleukin-6 were reduced after sulforaphane treatment indicating attenuated muscle damage and inflammation [18].

In the present study we applied a program of 7 consecutive days of exercise with High intensity interval training (HIIT) and twice daily administration of a glucosinolate rich sprout drink (GRS) or a placebo drink (PLA) in a double-blinded, placebo controlled cross-over fashion. The intent was to challenge the adaptive capacity and the antioxidative defence of the subjects and determine if administration of GRS in combination with exercise could activate Nrf2, enhance physical performance and protect against potential negative effects of excessive exercise.

## 2 Method

### 2.1 Preparation of sprouts

Broccoli raab sprouts and alfalfa sprouts (placebo) were grown in a commercial sprout growing facility (Munka-Grodden AB, Sweden). The sprouts were harvested on day 5 after initial soaking. After repeated washing, sprouts were homogenized in water with a ratio of 75 g of sprouts to 180 mL of water. The sprout drinks were then immediately frozen to −80° C. Upon consumption the drinks were quickly thawed, and 50 mL apple juice concentrate was added for improved taste and masking, together with 0.75 g brown mustard seed powder containing myrosinase [19] to facilitate the conversion of glucosinolates to isothiocyanates. Measurement of glucosinolates was conducted according to Mawlong et al. [20] with slight modifications. Freeze-dried sprouts were homogenized with 80% methanol in a 2 mL vial. The homogenate was directly centrifuged at 3000 rpm for 4 min at room temperature, and the supernatant was collected. 50 μL was used for the estimation, mixed with 50 μL MeOH and 2 mM (250 μL) STCP (58.85 mg Sodium tetrachloropalladate + 170 μL concentrated HCl + 100 mL dH_2_O). This was mixed thoroughly and immediately measured with spectrophotometry at 425 nm. The glucosinolate sinigrin was used as an internal standard. The broccoli sprouts contained 1.145 ± 0.035 mmol of total glucosinolates per 75-g fresh weight and the glucosinolate content in the alfalfa sprouts were 0.014 ± 0.001 mmol per 75-g fresh weight. The energy content of one dose of sprout drink including the apple juice was 90 kcal for the broccoli sprout drink and 84 kcal for the alfalfa sprout drink.

### 2.2 Sprout drink supplementation

The study design was a randomized, double-blinded, cross-over study with a 1-month wash-out between the different treatments. Subjects consumed 2 bottles of sprout juice per day, one in the morning and one in the evening. The bottles contained 75 g of sprouts each and the supplementation period continued for 9 days. The last GRS or PLA drink was consumed 78±4 min before the post-tests started. During the wash-out period, the subjects were instructed to follow their habitual diet and exercise pattern.

### 2.3 Standardization of diet

Subjects maintained their usual diet throughout the training week, specifically excluding glucosinolate-rich foods such as cabbage, kale, broccoli, and cauliflower. Participants were asked to keep a detailed food record, noting everything they consumed. This helped us to monitor adherence to the dietary instructions and assess any potential influence of uncontrolled dietary variations.

On the evening prior to pre- and post-tests, subjects consumed a standardized meal of 785 kcal (35 g fat, 98 g carbohydrates, 15 g proteins) provided by the research team. Subsequently, they fasted overnight, establishing a consistent pre-test nutritional state across all participants.

### 2.4 Training protocol

All nine subjects completed the seven training sessions in the intervention period. The sessions were self-paced, and the subjects were instructed to produce the highest mean power output during the part of the sessions that included repetitions of 4–8 min. Each 30-s sprint was performed in an all-out mode. The average power output during training was 204±27 W during PLA and 204±29 W during GRS. Average blood lactate at the end of the sessions were 11.7±2.3 mM in PLA and 11.0±2.0 mM in GRS (p = 0.28). The average heart rate during the training sessions was 168±10 bpm in PLA and 169±8 bpm in GRS (p = 0.58).

### 2.5 Pre- and post-test

Pre and post the training intervention, physiological characteristics during cycling were assessed using a SRM ergometer (Schoberer Rad Messtechnik, SRM, Julich, Germany). Submaximal work rate response was evaluated with 5-min intervals at fixed work rate and cadence, each separated by a minute of rest. Capillary blood lactate and glucose levels were measured and analysed with a Biosen C-Line Clinic. The initial work rate was set to 80–120 W, then increased by 15–30 W per stage until blood lactate accumulation >4 mM was observed. After a brief rest, an incremental maximal exercise test was conducted to establish VO_2_max. The test began at the previously achieved work rate, with 15–20 W increases per minute until fatigue. VO_2_max were determined from the highest average 30 and 45 s periods, respectively. Gas exchange was continuously measured using an Oxycon Pro device (Erich Jaeger GmbH, Hoechberg, Germany), heart rate was recorded (Polar Electro OY, Kempele, Finland) and perceived exertion rated at each stage via the Borg RPE-scale [21]. All equipment was calibrated as per manufacturers' instructions.

The pre-tests were done in the week before the training period started. After the last training session, subjects were allowed between 24 and 72 h of rest before the post-tests were done while they continued to consume 2 × 75 g of sprout drinks per day. The length of the rest was kept identical within subjects in the different conditions.

### 2.6 Muscle biopsies

Biopsies were collected from fasting subjects early in the morning after a 12-h rest period following the last HIIT session. Pre and post biopsies were taken from alternating legs in the vastus lateralis region using a randomized order among subjects. Local anesthesia was applied at the biopsy site before extracting roughly 100 mg of wet tissue using a needle biopsy. The samples were cleaned of visible fat, connective tissue, and blood, then divided into two parts: ∼ 50 mg for respirometric analysis in ice-cold ISO medium, and the other part was flash-frozen in liquid nitrogen for later analysis.

### 2.7 Mitochondrial respiration

Mitochondria were extracted from fresh muscle in isolation medium following Gnaiger and Kuznetsov's method [22] with modifications by Tonkonogi and Sahlin [23], and Larsen et al. [24]. The muscle was chilled, homogenized, then treated with bacterial protease, followed by centrifugation to separate and re-suspend pellets. Respiration of isolated mitochondria was gauged using a two-channel respirometer (Oroboros Instruments) in respiration medium MIR05 (EGTA 0.5 mM, MgCl_2_·6H_2_O 3 mM, K-lactobionate 60 mM, Taurine 20 mM, KH_2_PO_4_ 10 mM, HEPES 20 mM, Sucrose 110 mM, BSA 1 g L^−1^) at 37 °C, following manufacturers' calibration instructions. Respiration was assessed across various states using different combination of substrates including octanoyl carnitine 0.2 mM, malate 0.5 mM, pyruvate 5 mM ADP 2.5 mM, glutamate 10 mM, rotenone 0.5 mM, succinate 10 mM.

Protein content was analysed using spectrophotometric analysis and the Pierce 660 nm protein assay. Data were collected and analysed in DatLab 5.2 software.

### 2.8 Glycogen

Muscle glycogen was evaluated in roughly 2 mg of freeze-dried, dissected muscle following a slightly modified version of a previously described homogenization protocol [25]. In brief, all samples were first heated in 1 M KOH (70 °C for 20 min) and then incubated in NaAc + Hac and NaAc + amyloglucosidase (40 °C for 2 h). After a second incubation with reagents (20 min), the absorbance was read at 340 nm using a Beckman Coulter DU800 spectrophotometer.

### 2.9 Assessment of MDA and carbonyl content

Carbonyl content was determined in approximately 1 mg of freeze-dried muscle samples, dissolved in 1 M KOH and heated at 70 °C using Abcam Protein Carbonyl Content Assay Kit (ab126287). After DNPH and TCA were added to the samples, centrifugation was performed, and the pellet was dissolved in acetone. The mixture was blended and centrifuged again. The pellet was then dissolved in guanidine solution and added to a 96-well microplate for absorbance reading at 360 nm using a TECAN Infinite 200 Pro spectrophotometer. Protein content was then determined using Thermo Scientific Pierce Protein Assay Kit (22660) and measured at 660 nm.

Malondialdehyde (MDA) was evaluated using Sigma Aldrich Lipid Peroxidation Assay Kit (MAK085). The assay involves MDA's reaction with thiobarbituric acid (TBA) to form a colorimetric product indicative of MDA content. Homogenate containing about 1 mg freeze-dried muscle was added to MDA standard solution and BHT. After TBA solution was added and incubated at 95 °C for an hour, absorbance was measured at 532 nm in a Beckman Coulter DU800 Spectrophotometer.

### 2.10 Myeloperoxidase

Myeloperoxidase was measured in EDTA-plasma using the myeloperoxidase DuoSet ELISA kit (R&D Systems, Abingdon, UK) according to the manufacturer's instructions. All samples were diluted 1:100 in 1% BSA in PBS.

### 2.11 Continuous glucose monitoring

The participants were outfitted with a FreeStyle Libre™ continuous glucose monitor (CGM) sensor, Abbott Diabetes Care from Witney, UK. This device was strategically positioned on the deltoids posterior surface, adhering to the manufacturer's detailed guidelines. To ensure data integrity and minimize gaps in the data collection, participants received clear instructions to use the dedicated CGM reader for sensor scans at regular intervals, approximately every 8 h.

### 2.12 Insulin

The quantification of plasma insulin levels was carried out utilizing an ELISA kit (Invitrogen, KAQ1251), following the guidelines provided by the manufacturer.

### 2.13 Western blot

Freeze dried muscle was used for immunoblotting. The homogenization protocol has previously been described in detail [26] as well as the protocol and material for immunoblotting [27]. The Nrf2 antibody was purchased from Cell Signaling Technology (#12721), RRID:AB_2715528. The proteins from each subject were loaded on the same gels and in series, but in randomized order for treatment (PLA and GRS). The whole lanes on the memcode were used as a loading control. Molecular Imager ChemiDoc XRS system with Image Lab software (version 6.0.1; Bio-Rad) were used for visualization and quantification.

### 2.14 Statistics

A two-way analysis of variance (ANOVA) was used to assess the effects of a week of exercise combined with either GRS supplementation or PLA on various outcome measures. The two-way ANOVA allowed us to investigate both the main effects of the intervention (exercise) as well as the interaction effects between the different treatments. To control for multiple comparisons, we employed a post-hoc tests using the Holm-Sidak method. We also examined the main effects of the intervention and time on *a priori* specified parameters (Nrf2 expression and performance) to provide a comprehensive understanding of the data. In cases where pairwise comparisons were necessary to compare different groups, we utilized independent and paired sample t-tests, as appropriate. One subject failed to complete the maximal exercise pre-test during the placebo condition due to a vasovagal reaction and was excluded from the analysis in Fig. 2c–e. Values are mean ±S.E.M. p < 0.05 was considered significant.

## 3 Results

### 3.1 Skeletal muscle Nrf2 protein is modulated after GRS and exercise and protects against oxidative stress

**Fig. 1:**
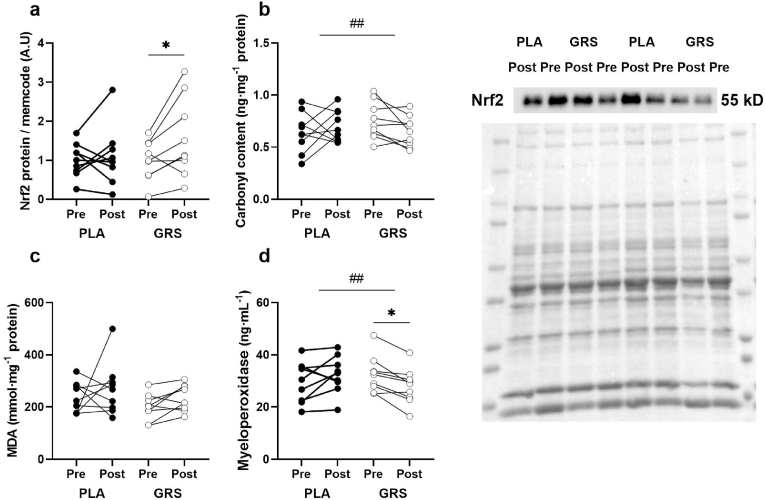
A) Shows total Nrf2 protein in whole muscle homogenate as assessed by Western Blot. Units are arbitrary units expressed per loaded protein as assessed by Memcode. Data are individual values. GRS = Glucosinolate-rich sprouts, PLA = Placebo, Pre = before the training and supplementation period, Post = after the training and supplementation period. A trend towards a main effect was found between pre to post (p = 0.09) but this effect was only significant in the GRS condition (p = 0.03, Holm-Sidaks post-hoc test). B) Carbonyl content in muscle homogenates assessed by ELISA. Units are ng per mg protein in the homogenate. C) Malondialdehyde content in muscle homogenates assessed by ELISA. Units are ng per mg protein in the homogenate. D) Myeloperoxidase content in plasma samples as assessed by ELISA. Units are ng per mL plasma. n = 9 in each condition. * = p < 0.05. ^##^ = p < 0.01.

We first wanted to understand if the intense exercise with GRS or PLA had increased the protein abundance of Nrf2 and activated the antioxidative defence system. We therefore measured Nrf2 in skeletal muscle homogenates using western blot technique. We found a near-significant main effect of training (p = 0.09) and a post-hoc test revealed that Nrf2 was significantly upregulated only in the GRS condition after exercise training (p = 0.03, pre compared to post) but not in the PLA condition (p = 0.83, pre compared to post, Sidaks post-hoc test). See Fig. 1a. Nrf2 activation is associated with induction of antioxidative genes, which should protect against exercise-induced oxidative stress. To assess whether GRS treatment also affected markers of oxidative stress we measured carbonyl content and malondialdehyde levels in the skeletal muscle homogenates. We found a significant interaction effect on carbonyl content between the GRS and PLA conditions. From pre to post exercise carbonyl content increased from 0.64±0.06 to 0.71±0.05 in the PLA condition and decreased from 0.75±0.06 to 0.65±0.05 in GRS (p = 0.03 by interaction), Fig. 1b. However, malondialdehyde levels were not changed both by either exercise or treatment (from 237±20 to 270±34 in PLA and from 200±17 to 234±17 in GRS, Fig. 1c). Thereafter, we measured myeloperoxidase concentrations in plasma samples to investigate if the altered redox status of the muscle cells was also reflected in the blood. Indeed, we found that myeloperoxidase increased from 29.6±2.6 to 32.4±2.4 in the PLA condition and decreased from 32.5±2.3 to 27.9±2.3 ng/mL in the GRS condition (p = 0.002 by interaction, Fig. 1d). Together these results indicate an increased antioxidative defence and a marked reduction in oxidative stress after GRS-treatment compared to PLA despite a more modest upregulation of Nrf2. Considering that the functional activation of Nrf2 is driven by its translocation into the nucleus, the modest elevation of Nrf2 observed here in whole muscle homogenates could indeed correspond to a substantial increase at the nuclear compartment, thereby having a more pronounced effect on antioxidant gene expression.

**Fig. 2:**
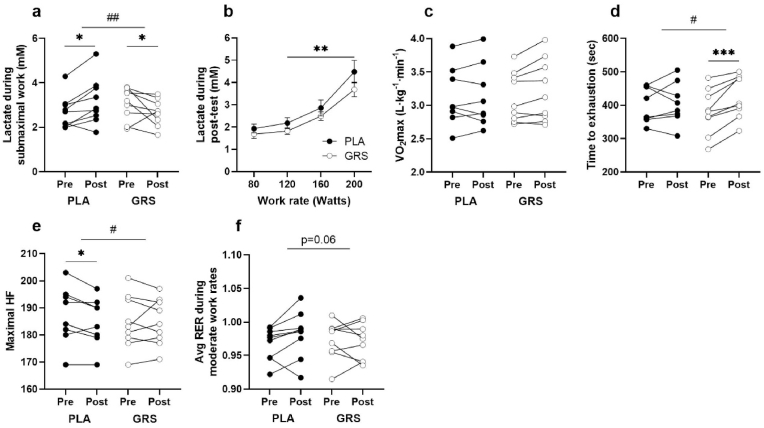
A) This graph represents the capillary lactate levels during submaximal exercise pre and post training in both the GRS and PLA conditions. n = 9 in each condition. B) Illustrates the improvement in work rate at an absolute lactate concentration post GRS or PLA administration. It depicts an approximate 15–20 W right-shift lactate concentration for a given work rate in the GRS condition compared to PLA. n = 9 in both conditions. C) Demonstrates the maximal oxygen consumption (VO_2_max) during an incremental exercise test to exhaustion in both the GRS and PLA condition pre and post training. No significant changes are observed in either condition. n = 8 in PLA, n = 9 in GRS. D) Shows the time to exhaustion during the maximal test for both the GRS and PLA groups. n = 8 in PLA, n = 9 in GRS. E) Represents the maximum heart rate measured at the end of the maximal incremental exercise test, pre and post the training period in both the GRS and PLA conditions. n = 8 in PLA, n = 9 in GRS. F) Depicts the respiratory exchange ratio (RER) at moderate work rates in both the GRS and PLA groups. n = 9 in each condition. Data are individual values. * = p < 0.05, **p < 0.01, ^#^p < 0.05 for interaction, ^##^p < 0.01 for interaction.

### 3.2 GRS decreases lactate accumulation during submaximal work and improves maximal performance

In animal studies, it has been demonstrated that the activation of Nrf2 enhances exercise performance [4,13]. A critical component of a physical performance improvement is the diminished accumulation of lactate during submaximal work, which indicates a primary adaptive response.

To investigate this further, we assessed capillary lactate levels during submaximal exercise. Notably, we observed a significant interaction effect where the lactate concentrations were markedly lower following the administration of GRS, as compared to PLA, under submaximal workload conditions. During submaximal work the average lactate concentration increased in PLA from pre 2.69 ± 0.24 to post 3.17 ± 0.35 mM and decreased in GRS from pre 3.06 ± 0.24 to post 2.66 ± 0.20 mM (p = 0.003 by interaction), see Fig. 2a. As can be seen in Fig. 2b, the work rate at an absolute lactate concentration was improved by 15–20 W or ∼15%. During an incremental exercise test to exhaustion maximal oxygen consumption (VO_2_max) was not significantly affected (3.12–3.14 L min^−1^ in PLA and 3.13–3.22 L min^−1^ in GRS, main effect p = 0.10, interaction effect p = 0.19, Fig. 2c). However, time to exhaustion during the maximal test was significantly improved in the GRS condition (from 401±19 to 406±22 s in PLA and 380±22 to 426±21 s in GRS, p = 0.02 by interaction, Fig. 2d). A well-known negative effect of intense periods of training is a reduction in maximum heart rate. Here we found maximum heart rate significantly decreased from 187±3 to 184±3 bpm in PLA but unchanged from 185±3 to 185±3 bpm in the GRS, p = 0.03 by interaction, see Fig. 2e.

A change in lactate concentrations during submaximal exercise could indicate a change in substrate preference. Therefore, we measured oxygen consumption (VO_2_) and carbon dioxide emission (VCO_2_) during submaximal work rate where the respiratory exchange ratio (RER) (VCO_2_/VO_2_) reflects substrate utilization. We found no main effects of the intervention but there was a tendency towards an interaction effect (p = 0.06) for a difference in RER at moderate work rates from 0.968±0.01 to 0.982±0.01 in PLA and from 0.973±0.01 to 0.971±0.01 (see Fig. 2f) possibly indicating a shift towards higher reliance on lipid oxidation after GRS compared to PLA.

### 3.3 Mitochondrial respiration and content are unchanged after GRS and PLA

**Fig. 3:**
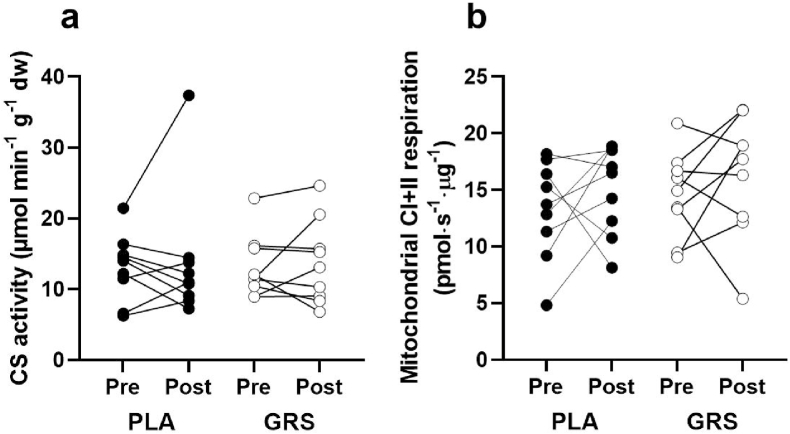
A) Displays the activity of citrate synthase in muscle tissue in pre and post training in both the GRS and PLA conditions. Units are in μmol min^−1^ g^−1^ dry weight. n = 9 in each condition. B) Illustrates the results of High Resolution Respirometry in isolated mitochondria for both the GRS and PLA groups. Mitochondria were activated with ADP, malate, glutamate, pyruvate and succinate at saturating concentrations. No significant main or interaction effects from the intervention are observed, suggesting that other mechanisms than an increased mitochondrial capacity may be responsible for the observed reduction in lactate. n = 9 in each condition.

Increases in mitochondrial capacity and function are expected outcomes of endurance exercise training. At a given submaximal work rate, a higher mitochondrial capacity should divert more pyruvate into mitochondrial oxidation instead of a reduction to lactate by lactate dehydrogenase. To understand if the lower lactate concentrations during exercise after GRS is due to an increase in mitochondrial content we assessed the activity of citrate synthase in the muscle tissue, but found it was unchanged in both conditions (from 6.5±2.4 to 6.9±4.6 in PLA and 6.6±2.3 to 6.9±3.0 μmol min^−1^ g^−1^ dw in GRS, (Fig. 3a). We also measured mitochondrial function by High Resolution Respirometry in isolated mitochondria but found no significant main or interaction effect by the intervention (Fig. 3b). Therefore, other mechanisms than an increased mitochondrial capacity must be responsible for the reduction in lactate.

### 3.4 GRS markedly attenuates nocturnal hypoglycaemia during periods with intense exercise training

**Fig. 4:**
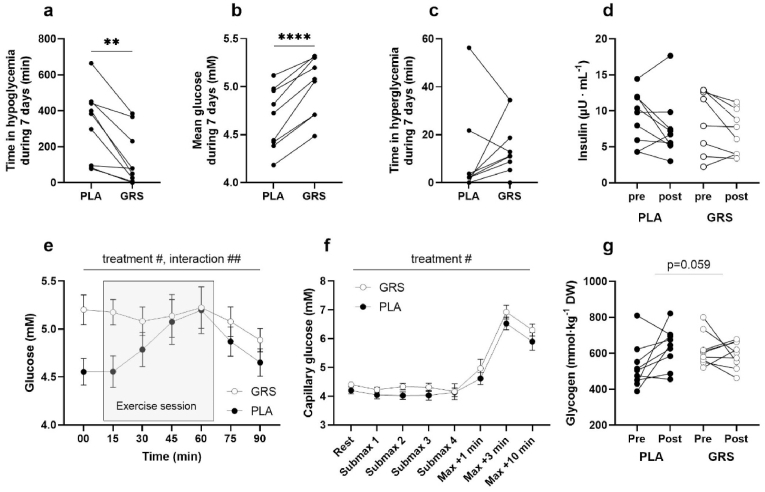
A) Daily duration of hypoglycemic episodes for the placebo (PLA) and glucoraphanin-rich sprouts (GRS) conditions. The average time with glucose levels below 4 mM was significantly reduced in the GRS (2 h 16 min ± 51 min) compared to the PLA (5 h 21 min ± 67 min). B) Comparison of 24 h mean blood glucose concentrations between the PLA and GRS groups. The mean glucose level was significantly lower in the PLA group (4.67±0.11 mM) than in the GRS group (5.02±0.10 mM). C) Daily duration of hyperglycemic episodes in the PLA and GRS groups. The time spent with glucose levels exceeding 8 mM was not significantly different between the PLA group (10±6 min) and the GRS group (16±4 min). D) Fasting insulin levels post-test for the PLA and GRS groups. There was no significant difference between the PLA group (7.5±1.4 mU L-1) and the GRS group (7.4±1.4 mU L-1). E) Glucose levels around each exercise session for the PLA and GRS conditions. The PLA group began with lower glucose levels, peaked towards the end of exercise, and then dipped to lower levels. The GRS group showed a slight elevation but maintained stable glucose levels throughout exercise. F) Glycogen concentration in muscle homogenates post-training in the PLA and GRS groups. A borderline significant interaction effect (p = 0.059) indicates an increase in glycogen levels in the PLA group, a trend not mirrored in the GRS group. n = 9 in each condition. Data are individual values. ** = p < 0.01, **** = p < 0.0001,_^#^p < 0.05, ^##^p < 0.01.

Intense exercise training periods seem to correlate with a higher incidence of both hypo- and hyperglycaemic episodes in elite athletes [6]. Sulforaphane has been demonstrated to normalize exaggerated hepatic glucose production and improve Hba1c in specific diabetic patient subgroups [28]. We aimed to corroborate these findings in our current study population, investigating whether GRS-rich sprouts could improve blood glucose control. Interstitial glucose was continuously monitored throughout the seven-day long training period, revealing that time spent with glucose below 4 mM averaged 5 h 21 min ± 67 min per day in the PLA group. In contrast, it decreased to 2 h 16 min ± 51 min daily in the GRS group (p = 0.002) Fig. 4a. Additionally, the mean blood glucose was significantly lower (4.67±0.11 mM in PLA versus 5.02±0.10 mM in GRS, p=<0.0001, Fig. 4b). Nonetheless, the time with blood glucose exceeding 8 mM was similar in both groups (10±6 min in PLA and 16±4 min in GRS per 24 h, Fig. 4c). Despite the substantial changes in CGM readings, the fasting insulin levels at the post-tests were unchanged between conditions (7.5±1.4 mU L^−1^ in PLA, and 7.4±1.4 mU L^−1^ in GRS, p = 0.92, *t*-test, Fig. 4d). The CGM readings represents weekly average readings across all subjects. However, given that meals and training sessions took place at slightly different times each day throughout the week, this approach might overlook subtle variations in the data. We therefore looked more specifically at the glucose response at the time around each exercise session. We discovered a significant interaction effect that pointed to distinct glucose responses under the GRS and PLA conditions. During exercise, the glucose level in the GRS group was slightly elevated but remained stable. In contrast, the PLA group started with a lower glucose level, peaked to match the GRS group towards the end of the exercise, but then dipped to a lower level again (Fig. 4e). Statistically, these variations were significant; treatment effect p = 0.04 and interaction effect p = 0.001. Given the potential differences between glucose levels measured in the interstitial fluid and in the blood, we sought to validate our findings using capillary blood measurements taken during the post-test. Although these tests were performed 24–72 h after the last training session, we observed a significant treatment effect, characterized by marginally elevated blood glucose levels during submaximal exercise and following the maximal exercise test (p = 0.02 by treatment effect, Fig. 4f). This in-depth analysis underscores the differential impact of the GRS and PLA treatments on glucose dynamics during exercise.

The findings regarding altered glucose and lactate levels in blood could indicate that GRS treatment may influence the synthesis or utilization of glycogen in skeletal muscle. This, in turn, could contribute to the observed changes in glucose and lactate dynamics. Notably, the primary source for lactate formation in skeletal muscle is muscle glycogen. As such, the lower capillary lactate levels observed during exercise might serve as an indicator of decreased glycogen concentrations. We thus proceeded to assess muscle glycogen in muscle homogenates. Our findings revealed a borderline significant interaction effect (p = 0.059). As illustrated in Fig. 4g, there was an observable increase in glycogen post-training within the PLA group, a trend not mirrored in the GRS group. This adds a layer of complexity to our understanding of the impact GRS treatment has on metabolic processes.

## 4 Discussion

Here we show, that incorporating supplementation with glucosinolate (GRS)-rich broccoli sprouts into a 7-day intense training regimen in a cohort of healthy subjects not only mitigates several markers of oxidative stress and improves the blood glucose profile but also enhances the exercise-induced adaptations observed in the participants.

We also observed a reduction in carbonylated proteins within muscle tissue and myeloperoxidase levels in the blood following one week of GRS administration and training. This indicates that the overall adaptations to acute oxidative stress induced by exercise and GRS were towards diminished oxidative stress and improved physical performance. In contrast, oxidative stress increased during PLA-condition with blunted physiological adaptations.

Human exercise studies utilizing glucosinolate or isothiocyanate administration are sparse. However, in a recent study by Komine et al., it was demonstrated that a two-week regimen of sulforaphane supplementation prior to engaging in eccentric exercise effectively mitigated both delayed onset of muscle soreness and markers of oxidative stress in human subjects. Notably, this was accompanied by an increase in NQO1 mRNA expression in blood samples [17]. Similarly, Oh et al. discovered that sulforaphane injections in wild-type mice significantly bolstered running endurance. This improvement was attributed to the upregulation of Nrf2 signaling and the mitigation of oxidative stress-induced muscle damage during exhaustive exercise, thereby highlighting the potential role of sulforaphane in augmenting exercise performance [13].

Although the beneficial effects of moderate exercise are well-known, periods with excessive exercise have been shown to have negative effects on both muscle and metabolic function. In a previous investigation, we demonstrated the emergence of reduced glucose tolerance and loss of mitochondrial function following a three-week progressively intensified training period [6]. Building upon these findings, the current study employed a comparable training protocol, specifically focusing on the final week of the three-week period. Surprisingly, despite the implementation of this rigorous training week, we did not observe a reduction in mitochondrial function, irrespective of the administered supplementation indicating that the training load was well accepted by our subjects.

The markedly reduced hypoglycemia in the present study suggests that factors beyond carbohydrate intake and energy balance, such as oxidative stress, might play a pivotal role in glucose regulation. This highlights the complexity of metabolic responses to strenuous exercise and indicates the need for further investigations into the relative contributions of energy balance and oxidative stress to glucose homeostasis in athletes.

An unexpected finding in this study was that blood lactate concentrations during submaximal exercise were lower after GRS compared to PLA. The lower lactate accumulation often seen after a period of training is often attributed to either an increased oxygen delivery or improved mitochondrial capacity. We did not find any significant differences in either VO_2_max or mitochondrial function or capacity, indicating that other, unknown mechanisms were at play.

The observed decrease in lactate levels may have broader implications beyond the scope of exercise physiology. Isothiocyanates, for instance, are postulated to exhibit chemo preventive properties and have demonstrated inhibitory effects on cancer development across various cellular- and animal models [29]. Singh et al. recently demonstrated that sulforaphane effectively inhibited cancer cell proliferation by suppressing glycolysis and consequently diminishing the activity of several glycolytic enzymes. This led to a consequential decrease in lactate accumulation. However, when resting plasma lactate levels in prostate cancer patients were analysed post-administration of broccoli sprout extract, no significant reductions in lactate levels were detected [30].

The observation of increased interstitial glucose throughout the day, as measured by continuous glucose monitors, coupled with decreased lactate levels is intriguing. One plausible explanation for this pattern could be enhanced gluconeogenesis, leading to increased conversion of lactate to glucose.

Alternatively, the observation might be attributed to an upregulation in the reverse reaction of lactate dehydrogenase, where lactate is converted back to pyruvate. This would subsequently make more pyruvate available for further oxidation in mitochondrial metabolism. Our data, which indicated a trend towards a lower respiratory exchange ratio (RER) during submaximal exercise and a less pronounced increase in glycogen following GRS administration, supports this hypothesis. These findings suggest a decreased reliance on glycogen as a metabolic substrate following the consumption of GRSs.

In summary, supplementation with GRS in combination with intense exercise training seems to ameliorate oxidative stress and improve the physiological adaptations to the training program with lower lactate concentrations during exercise and attenuated time spent in the hypoglycemic range. The mechanism behind these novel findings is subject to future investigations.

## Funding

This study was funded by grants from Ekhagastiftelsen, 10.13039/501100005350Swedish Research Council for Sport Science and Sydgrönt Ekonomisk Förening.

## Declaration of generative AI and AI-assisted technologies in the writing process

During the preparation of this work the authors used MS Word text prediction and Open AI services in order to check grammar and language. After using these services, the authors reviewed and edited the content as needed and take full responsibility for the content of the publication.

## Declaration of competing interest

This study was partially sponsored by Sydgrönt, an economic association for vegetable growers. The sprouts used in the study were generously provided by Munka-Grodden AB, a company specializing in the commercial cultivation of sprouts. These organizations did not play any role in the conduct, analysis, or interpretation of the study's data. Authors FJL and MLS hold a patent related to the use of isothiocyanates for enhancing adaptations to exercise training. In addition, they are co-founders and shareholders in a commercially operational entity, which aims to market and sell products designed to augment athletic performance. No other authors have any conflicts of interest to declare.

## Data Availability

Data will be made available on request.
